# Biosimilar Use in Breast Cancer Treatment: A National Survey of Brazilian Oncologists' Opinions, Practices, and Concerns

**DOI:** 10.1200/GO.20.00649

**Published:** 2021-08-20

**Authors:** Heloísa M. Resende, Leandro Ladislau, Ana Carolina F. Cardoso, Juliana Dinéia P. Brandão, Biazi R. Assis, Paola Cardoso, Pedro Henrique A. Marassi, Vivienne Castilho

**Affiliations:** ^1^ Hospital Hinja, Volta Redonda, Rio de Janeiro, Brazil; ^2^ Departamento de Clínica Médica, Centro Universitário UNIFOA, Volta Redonda, Rio de Janeiro, Brazil; ^3^ Libbs Farmacêutica, São Paulo, São Paulo, Brazil; ^4^ Departamento de Clínica Cirúrgica, Centro Universitário UNIFOA, Volta Redonda, Rio de Janeiro, Brazil

## Abstract

Breast cancer is the most common malignancy in Brazilian women, with 66,280 new cases in 2020 (with 20% overexpressing human epidermal growth factor receptor 2 [HER2]). The trastuzumab biosimilar was the first oncology biosimilar approved in Brazil for HER2-positive breast cancer treatment. This study aimed to assess the current level of knowledge of biosimilars, comfort of use, extrapolation indications, and switching of practices among oncologists in Brazil.

**METHODS:**

A 24-question survey was developed using an online platform that sought information regarding responders' characteristics and use of biosimilars. The survey analyzed the basic knowledge of biosimilars, trastuzumab biosimilars, level of comfort with extrapolation, switching treatment regimens, and opinions concerning the cost of HER2-positive breast cancer therapy. Data were collected between July and September 2019 and included 144 oncologists from five Brazilian regions.

**RESULTS:**

In total, 95% of respondents could identify the most appropriate definition of biosimilars and 96% felt comfortable prescribing trastuzumab biosimilars. Although 63% of respondents would use the biosimilar in all settings wherein the reference biologic was approved, 35% would use the biosimilar for cases involving metastatic disease. Although 82% of oncologists were in favor of switching from a reference biologic to a biosimilar, 18% would avoid switching regimens. The lack of studies detailing switching to other regimens and the correct timing to switch was the major concern. The cost of HER2 therapy was a significant concern for most oncologists.

**CONCLUSION:**

Oncologists demonstrated a high level of knowledge of biosimilars and encouraging levels of prescriber use; however, extrapolation and switching treatment regimens are barriers to the effective use of biosimilars in cancer treatment. Efforts should be concentrated on strategies involving medical education programs on biosimilars.

## INTRODUCTION

The US Food and Drug Administration (FDA) and European Medicines Agency (EMA) define a biosimilar as a biologic molecule product that is highly similar to and has no clinically meaningful differences from an existing approved reference product.^1,2^ Its similarity to the originator biologic is established by means of comparability studies, which are a comprehensive head-to-head comparison of the biosimilar with the reference product to demonstrate high similarity in chemical structure, biologic function, efficacy, safety, and immunogenicity.^3-5^

**Context**

**Key Objective**
Physicians have different perceptions regarding the uptake of biosimilars, and a good understanding of biosimilars does not automatically translate into prescription. Considering the discrepancies in some concepts around biosimilars and their regulatory designation among different countries, to our knowledge, this was the first study in Brazil to describe oncologists' opinions, practices, and concerns regarding biosimilars and trastuzumab biosimilars.
**Knowledge Generated**
Our data demonstrated a good understanding and acceptance of biosimilars by prescribers. Moderate knowledge of clinical studies supporting approval, level of comfort in extrapolation, and concerns about switching treatment regimens appeared among the main aspects that require further research.
**Relevance**
Future educational initiatives among Brazilian oncologists could contribute to a broader understanding of concepts involving biosimilars and the extrapolation of indications. Adherence to a treatment regimen involving biosimilars could broaden access to high-cost treatments represented by biologic drugs, especially for patients with cancer.

In Brazil, the National Health Surveillance Agency (ANVISA) has developed guidelines for evaluating oncology biosimilars through Collegiate Board Resolution No. 55,^6^ which is based on accepted international standards, such as those of the WHO^7,8^ and also meets most of the policy reviews on oncology biosimilars.^9-11^ According to this resolution, innovative biologic products may follow the innovative pathway and biosimilar products may follow the comparability pathway. Following EMA and FDA, ANVISA requires an extensive comparison of the biosimilar with the reference product to demonstrate high similarity in chemical structure, biologic function, efficacy, safety, and immunogenicity.^2,6^

Trastuzumab reference (Herceptin; Hoffmann-La Roche Ltd, Kaiseraugst, Switzerland) was first approved in 1999 by ANVISA in Brazil, and the trastuzumab biosimilar was the first oncology biosimilar approved in 2017 for human epidermal growth factor receptor 2 (HER2)–positive breast cancer and advanced gastric cancer treatment.^12^ Trastuzumab is a monoclonal antibody against HER2, which promotes an increase in survival of patients diagnosed with HER2-positive breast cancer.^13^ To date, other three brand trastuzumab biosimilars have also been approved by ANVISA;^14,15^ however, before 2017, patients were only offered trastuzumab reference.

The main advantages of biosimilars include their lower prices compared with the reference drug.^16^ However, acceptance and/or adhesion to biosimilars has many challenges. Previous survey findings have demonstrated the prescribers' concerns and doubts about the biosimilars approval process, definition of interchangeability or switching and their rules, requirements for extrapolation, and safety and efficacy.^17-19^

Extrapolation refers to the extension of clinical data for reference products to biosimilars, since both reference and biosimilars have the same mechanism of action.^11^ Extrapolation is a regulatory term on the basis of comparative pathways, including phase I pharmacokinetic and pharmacodynamic studies and phase III trials assessed on a case-by-case basis. Although a biosimilar gains regulatory approval for extrapolation considering the comparative pathway, the surveys have demonstrated a broad range of levels of acceptability and knowledge about this concept.^18,19^ Interchangeability is also a regulatory term that characterizes two medical treatments that are therapeutically equivalent and can be safely switched in clinical practice. Switching refers to the clinical action, that is, when the prescriber decides to exchange one drug for another.^11^ Interchangeability is regulated by different national regulatory authorities in European Union, United States, and other countries. In the United States, FDA has created a regulatory designation pathway for the scientific evaluation of interchangeability, whereas in European Union, the responsibility for conferring interchangeability designation is delegated to individual member countries.^11^ In Brazil, ANVISA states that the demonstration of interchangeability shall not be a regulatory requirement for the registration of a biosimilar and emphasizes that medical evaluation is essential in the case of switching from reference to biosimilar.^20^

A recent systematic review covered 23 studies that collected physicians' perceptions regarding the uptake of biosimilars. The authors showed a wide variation in physicians' self-rated knowledge of biosimilars: 2%-25% did not demonstrate any understanding about biosimilars and 18%-66% of physicians incorrectly described biosimilars as generic drugs.^21^ The European Society for Medical Oncology (ESMO) also conducted a survey in 2017 to assess physicians' perceptions about biosimilars, and they found encouraging levels of prescriber use; however, they identified some gaps in knowledge including biosimilar development, clinical trial design and end point selection, and requirements for extrapolation.^19^ In summary, a positive attitude related to the biosimilars does not automatically translate into prescription.

Considering the discrepancies in some concepts around biosimilars and their regulatory designation among different countries, as demonstrated by Sarnola et al^21^ and Giuliani et al,^19^ we proposed this study to examine Brazilian oncologists' knowledge and concerns regarding the uptake of biosimilars and trastuzumab biosimilars in Brazil.

## METHODS

### Study Design

The study comprised a 24-question survey using the IQVIA online platform through computer-assisted web interviewing, a widely used technique to apply customized online surveys, and sought information regarding responders' characteristics, responders' use and basic knowledge of biosimilars, level of comfort with extrapolation, switching of treatment regimens, and opinions concerning the cost of HER2-positive breast cancer therapy.

The survey questions were piloted with a small group of oncologists to test content, ease of understanding, and acceptability. The final online questionnaire was administered to oncologists' members of the MOC group (Brazilian Manual of Clinical Oncology), an educational platform with exclusive access to oncologists and hematologists. Oncologists were eligible if they have prior clinical practice experience in HER2-positive breast cancer and consented to data collection. The study was approved by the Ethics Committee of Fundação Oswaldo Aranha—UNIFOA (No.: 14816919.5.0000.5237).

Data were collected between July and September 2019 and included 144 oncologists representing five Brazilian regions: north, northeast, center-west, southeast, and south. The number of oncologists from each region was predetermined on the basis of the distribution and representativeness of all oncologists in Brazil, according to the IQVIA sampling methodology. The questionnaire was administered following the oncologists' consent and agreement to participate in this survey. The 144 oncologists who participated in the survey were compensated by IQVIA.

Oncologists were invited to participate via e-mail, which contained a link to the survey. The survey comprised 13 questions about demographics, medical training, and practice information and 11 questions assessing the study participant's knowledge of biosimilars. The study participants were then directed to the trastuzumab biosimilar use (Data Supplement).

### Statistical Analysis

The survey contained a mixture of checkbox answers and one question asking the responders to rank their level of agreement with each statement from 1 to 10. Respondents were not allowed to skip questions, and the survey was finalized when all questions were answered. The defined margin of error for the 144 respondents was 8%. Sampling continued until our quota (144 oncologists met the eligibility criteria) was reached, and we only considered respondents who fully completed the questionnaire. The results are summarized using descriptive statistics. The frequencies and proportions of all the categorical data were calculated. Bar plots and stacked bar plots were used to visualize the data.

## RESULTS

### Participant Demographics

The median age of the respondents was 38 years (range 36-45 years), and the majority of respondents were male (55.6%). Most of the respondents (70%) had between 3 and 10 years of clinical practice experience, whereas 30% had more than 10 years of clinical practice experience. The median time of clinical practice experience was 9 years. The time dedicated to direct patient assistance was 83.4%, whereas 8.9% of respondents expended their time on academic activities, 5.2% on administrative activities, and 2.1% on other activities. Each respondent assisted, on average, 18.3 patients with HER2-positive breast cancer per month. Regarding distribution of time between health care systems, considering the total number of respondents, 70% of the respondent's time is dedicated to private assistance, whereas 30% of their time is dedicated to the Brazilian unified health system (Sistema Único de Saúde [SUS]). The characteristics of the oncologists are presented in Table 1.

**TABLE 1:**
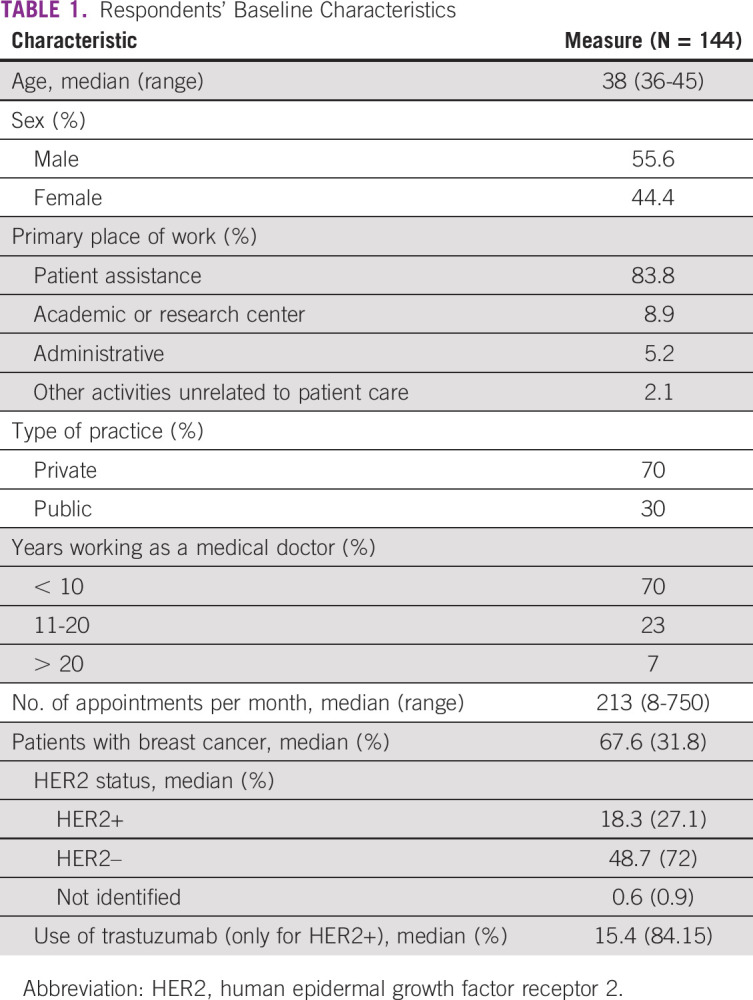
Respondents' Baseline Characteristics

### Knowledge of Biosimilars and Biosimilar Trastuzumab

**FIG 1:**
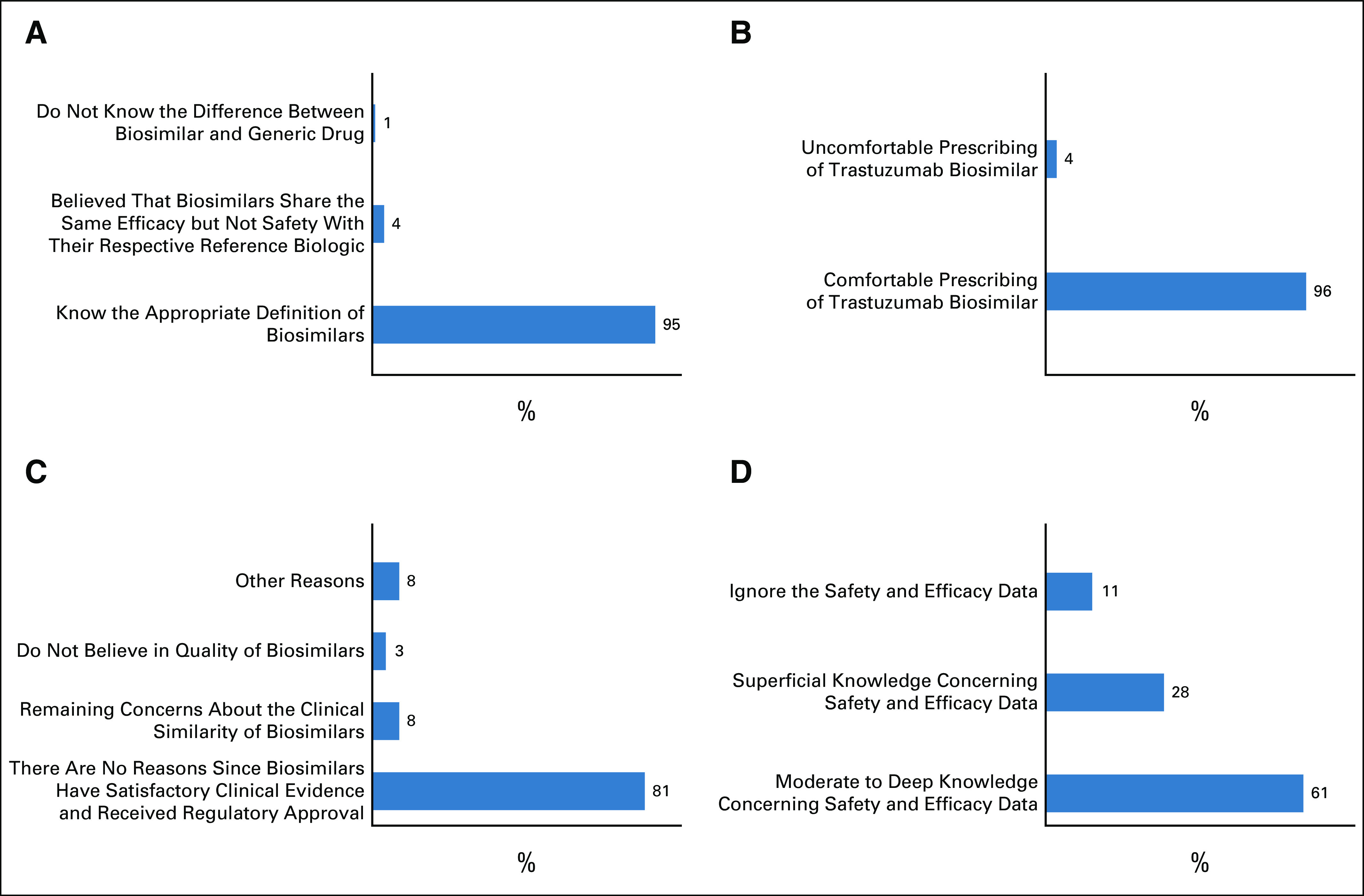
(A) Level of knowledge of the appropriate definition of biosimilars. (B) Comfort level of routine prescription of biosimilars by oncologists. (C) Reasons that could inhibit or interfere with the prescription of trastuzumab biosimilar. (D) Level of knowledge of Heritage study and the trastuzumab biosimilar development process.

Of the three options, 95% of respondents identified the most appropriate definition of biosimilars, 4% believed that biosimilars share the same efficacy but not safety with their respective reference biologic, whereas only 1% identified biosimilars as a generic drug (Fig 1A). In addition, 96% felt comfortable prescribing trastuzumab biosimilars, as long as the biosimilars demonstrated efficacy and safety similar to that observed in the trastuzumab reference, and 4% felt uncomfortable because they were unaware about the rules concerning the biosimilar approval (Fig 1B).

Regarding the reasons that could inhibit or interfere with the prescription of trastuzumab biosimilars, 81% of oncologists do not have concerns regarding the same and have already incorporated biosimilars in their routine clinical practice. However, 8% of oncologists still have concerns about the clinical similarity of biosimilars, for example, the lack of knowledge about the evidence of safety and efficacy. Moreover, 3% did not use or believe in the quality of biosimilars and 8% said that they had other reasons not mentioned before (Fig 1C).

When analyzing their knowledge of the Heritage study, which was a phase III study that supported the trastuzumab biosimilar approval in the United States, Europe, and Brazil, we observed that 61% of oncologists had moderate to deep knowledge concerning safety and efficacy data and 28% had superficial knowledge regarding the topic. It was observed that 11% had already heard about the study; however, they ignored the safety and efficacy data (Fig 1D).

### Extrapolation of Indications

**FIG 2:**
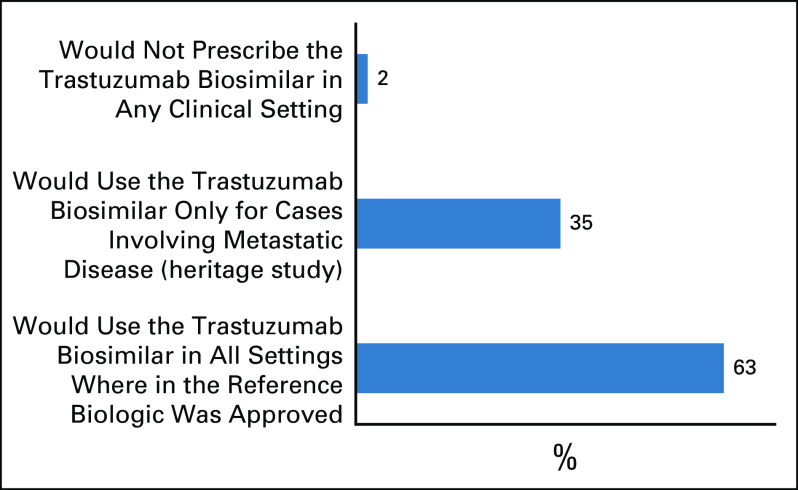
Oncologists' responses when asked if they would prescribe trastuzumab biosimilar in extrapolated indications wherein the reference biologic is approved for use.

When asked if they would prescribe trastuzumab biosimilar for all indications approved in the label (neoadjuvant or adjuvant setting), a majority of oncologists (63%) answered that they would use the biosimilar in all settings wherein the reference biologic was approved; 35% answered that they would use the biosimilar only for cases involving metastatic disease of the Heritage study, and 2% would not prescribe the biosimilar in any clinical setting (Fig 2).

### Switching Practice Among Oncologists

**FIG 3:**
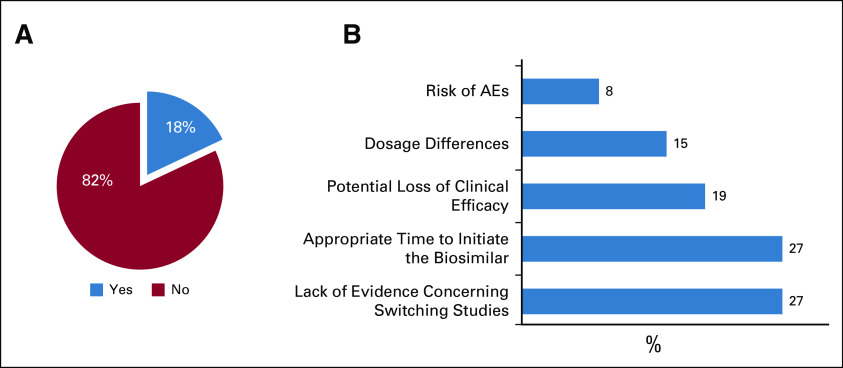
(A) Restrictions concerning switching a patient's treatment from a reference biologic to a biosimilar. (B) Major oncologists' concerns regarding switching treatment regimens. AE, adverse event.

Regarding switching a patient from a reference biologic to a biosimilar, 82% of oncologists had no restrictions concerning this practice (Fig 3A). However, 18% did avoid considering switching to the best possible extent, and the majority of concerns were expressed because of the lack of evidence concerning switching studies (27%), the appropriate time to initiate the biosimilar (eg, only in biologic-naïve patients) (27%), potential loss of clinical efficacy (19%), dosage differences (15%), and the risk of adverse events (AEs) (8%) (Fig 3B).

Furthermore, 68% of respondents agreed that the patients must be informed about the decision to use a biosimilar in their treatment. Besides communicating with the patients, 33% of oncologists would also share the decision with them. However, 32% of respondents believed that such a decision was irrelevant to patients and preferred not to inform them.

### Oncologist's Opinion Regarding Pharmacovigilance and Prescription Decision

Most respondents (67%) considered pharmacovigilance as very important, and they ensured their patients’ engagement in pharmacovigilance programs. Despite considering the importance of pharmacovigilance, 19% reported delegating responsibility to the multidisciplinary team and 8% did not encourage patients' participation. About 7% answered that they ignored a pharmacovigilance program.

**FIG 4:**
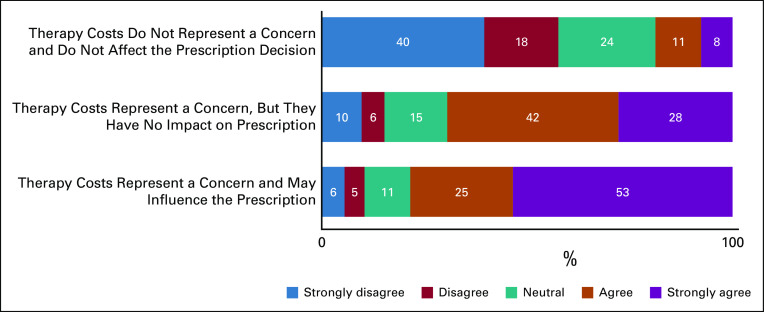
Prescribers' responses rating their level of agreement on a scale of 1 (strongly disagree) to 10 (strongly agree) among three statements regarding the influence of HER2-positive therapy cost on prescription practices. HER2, human epidermal growth factor receptor 2.

Respondents rated their level of agreement in three statements about the costs of HER2-positive breast cancer therapy and whether it would affect their prescription practices (Fig 4). The results are summarized on a five-point scale: strongly disagree (1-2), disagree (3-4), neutral (5-6), agree (7-8), and strongly agree (9-10). Only 8% of oncologists strongly agreed that therapy costs do not represent concerns and do not affect prescription decisions. Therapy cost represents concerns for treatment access as 53% of respondents strongly agreed that it may influence their prescription, since all treatment options have similar safety and efficacy, and 28% reported that despite the concerns, it has no impact on prescription decision.

Finally, we analyzed those who decided to prescribe anti-HER2 therapy within the private institution where the respondents worked. Overall, 32% of oncologists hold the decision to prescribe; 50% said that the decision is shared between physicians and the administrative sector; and in 18% of the sites, the administrative sector is exclusively responsible for making the decision.

## DISCUSSION

Biosimilars are important options to broaden access to high-cost treatments represented by biologic drugs, especially for patients with cancer.^16^ However, within the medical community, adherence to a treatment regimen involving biosimilars has many limitations, primarily because of concerns regarding safety, immunogenicity, extrapolation, and switching treatment regimens.^21,22^

Among the oncologists interviewed in our study, 95% correctly identified the definition of a biosimilar, although generic medicine was among the options. The percentage reported in the present study was higher than the percentage reported in a similar study conducted by Chapman et al^18^ in 2017, which showed that among UK prescribers, 72% correctly identified the concept. In line with the UK findings, another study with a similar methodology to the present study, conducted by ESMO in 2017 revealed that 74.6% of EU oncologists correctly identified the most appropriate definition of biosimilars.^19^ Sarnola et al pointed out that familiarity with biosimilars might be correlated with physicians' years of clinical practice experience and specialization. In fact, the median time of clinical practice was 9 years among Brazilian respondents, and all of them had a specialization in oncology.^21^

In line with knowledge about biosimilars, the percentage of biosimilar acceptance among Brazilian oncologists was high, with 81% stating that there were no reasons that prevented them from prescribing biosimilars. This good acceptance might be influenced by previous clinical experience with biosimilars for supportive drugs in oncology such as filgrastim and epoetin, which were approved in 1994 and 1996, respectively, before ANVISA's regulation for the approval of biosimilars was released in 2010.^23,24^ However, respondents were given restricted options for this question, relying on reasons about quality, safety, and efficacy and excluding issues about extrapolation, switching, or administrative reasons, such as the unavailability of biosimilars in their workplace or the inability to decide the prescription. According to our data, 70% of respondents dedicate their time to private assistance and only 32% hold the decision to prescribe.

Our data suggest that the majority of oncologists would prescribe trastuzumab biosimilars (96%); however, only some of them (63%) would extrapolate from metastatic disease to adjuvant or neoadjuvant settings. These results could reflect a gap in Brazilian prescribers' knowledge of the scientific justification underlying the regulatory approval of biosimilars, as 37% of the respondents viewed extrapolation as a problem even in the same disease setting. We did not address questions about extrapolation from one disease to another. Similar results were found in the European survey, which demonstrated that 57.4% of the prescribers would use an EMA-approved biosimilar and 76.7% would prescribe biosimilars for an extrapolated indication.^19^

Regarding the practice involving switching of regimens, 82% of the interviewees answered that they had no restrictions concerning switching the treatment from the reference to the biosimilar drug. The percentage obtained in the present study differs from those obtained in studies from Cohen et al and Chapman et al, which can be attributed to differences among prescribers' specialization in oncology and rheumatology, respectively.^17,18^ The survey conducted by ESMO, which was addressed for prescribers specialized in oncology, indicated that a majority of prescribers agreed that switching treatment regimens will have no significant effect on the treatment benefit.^19^ However, the majority of concerns expressed were regarding the potential for AEs and increased risk of immune reactions when switching treatment regimens.^19^

To date, no consensus exists among stakeholders regarding switching practice and the current data available mainly detail practices in rheumatology. Evidence from randomized controlled trials and real-world data has supported the efficacy and safety of a single or multiple switching for biosimilars of infliximab, etanercept, and adalimumab.^25-28^ In oncology, few switching studies support the comparative safety and efficacy of biosimilars of rituximab,^29,30^ trastuzumab,^31^ and bevacizumab.^32^ However, available data do not suggest that such a switch will result in significant loss of efficacy or increased AEs or immunogenicity. A recent systematic review analyzed 178 switch studies for 11 biosimilars, including one for trastuzumab. Different study designs were identified, but on the basis of the conclusions of the authors, most of them did not identify major safety, efficacy, or immunogenicity issues because of the practice of switching treatment regimens.^33^

Careful postmarketing pharmacovigilance should be conducted for all biopharmaceuticals, both reference and biosimilars, as these types of data provide additional evidence to guide clinical practice. Here, respondents considered pharmacovigilance to be very important, dedicating themselves personally or through the guidance of a multidisciplinary team to ensure that their patients are aware of the importance of reporting AEs. Active pharmacovigilance has also been cited as a motivator for biosimilar prescriptions.^22^ Since 2013, the EU has labeled biosimilar oncologic drugs with a black triangle for monitoring long-term AEs.^11^ In Brazil, patients and health care professionals are also encouraged to submit all AEs related to biologic drugs, despite the products not having a black triangle on the label. However, ANVISA and EMA require the inclusion of the Risk Management Plan for innovative biologic products and biologic products registered following the comparability pathway.^34,35^ Moreover, an active pharmacovigilance program to support patients receiving trastuzumab biosimilar treatment was sponsored by the manufacturer to closely monitor the AEs in Brazil. Two abstracts presented at the American Society of Clinical Oncology 2019 and ASCO 2020 Congresses reported no new safety signals detected or any differences in safety data between the biosimilar and the trastuzumab reference.^36,37^

In agreement with previous surveys,^18,21,38^ 73% of respondents indicated that costs are an aspect considered in medical decision making. Biosimilars have potential to transform costs in oncology considering the large volume of expenses related to the use of biologics.^10^ The most recent Policy Review in oncology biosimilars addressed price discounts for biosimilars, which were reported to be the average of 30% in EU and 10%-33% in the United States.^11^ Giuliani and Bonetti^39^ assessed the pharmacologic costs of trastuzumab reference and biosimilar to be necessary to get the benefit in neoadjuvant breast cancer treatment. Combining the costs of therapy with the measure of efficacy (pathologic complete response), the costs of the trastuzumab biosimilar is about 40% less compared with that of the trastuzumab reference (3,283€ and 6,310€, respectively) for the whole neoadjuvant treatment. In Brazil, the high costs of biologic drugs represent a challenge, for example, patients were granted access to the trastuzumab reference in the public health system 13 years after its approval.

To our knowledge, this was the first study in Brazil to describe and analyze oncologists' opinions, practices, and concerns regarding trastuzumab biosimilars. Although sample size was an important limitation of this study, as it represents approximately 4% of Brazilian oncologists, our data demonstrated a good understanding and acceptance of biosimilars by prescribers. Moderate knowledge of clinical studies supporting approval, level of comfort in extrapolation, and concerns about switching treatment regimens appeared among the main aspects that require further research. This raises the opportunity for future educational initiatives to contribute to a broader understanding of concepts involving biosimilars and the extrapolation of indications.
